# The impact of a student-developed, student-marketed, student-implemented and student-led 8-week health and wellness program on faculty and staff participation consistency

**DOI:** 10.3389/fpubh.2025.1659127

**Published:** 2025-10-14

**Authors:** Jeffrey Allen, Charles Olomofe, Shane Lehman

**Affiliations:** ^1^Department of Public Health and Health Science, St. Bonaventure University, St. Bonaventure, NY, United States; ^2^Department of Health, Physical and Secondary Education, Fayetteville State University, Fayetteville, NC, United States

**Keywords:** faculty and staff, student-driven, health and wellness, nutrition, consistency, fitness, participation

## Abstract

College and university faculty and staff in the United States experience diabetes rates higher than the national average, elevated cholesterol levels, receive fewer than the recommended 7 h of sleep per night, and routinely experience overwhelming anxiety. In response, some universities have implemented top-down approaches to employee wellness but there is scant evidence of student-driven approaches to faculty and staff wellness. The current study examines improvement in faculty and staff participation consistency when enrolled in a fully student-developed, −implemented, and -evaluated 8-week health and wellness program. The 78 participants in the study were asked to complete both a pre- and post-intervention Likert scale questionnaire, which was assessed by independent T-tests. There was a statistically significant difference in mean of the participation of staff in the wellness program activities within the university and at local community activities pre- and post-challenge (Mean difference: −0.456, 95% CI: −0.882 – −0.048; *p* = 0.029). In a similar vein, there was a statistically significant difference between pre-intervention and post-intervention participation in nutritional programs at the university and the surrounding local community by staff and faculty (Mean difference: −0.472, 95% CI: −0.817 – −0.127; *p* = 0.008). This increased participation could be the result of nudging used throughout the intervention, which included weekly consistency reporting reminders, weekly newsletters, regular email promotions, mailbox stuffers, and personal reminders to participate. It is our belief that this nudging approach led to habituation of participation amongst faculty and staff members.

## Introduction

### National burden of disease

Some of the most prevalent chronic diseases in the United States include heart disease, diabetes, cancer, and stroke (1). Chronic disease is defined as conditions lasting more than one year that require regular medical care and affect the activities of daily living (2). Chronic diseases are often the result of poor health behaviors, exacerbating health conditions, and endemic socioeconomic and environmental effects. Some of these health behaviors and conditions include limited physical activity, poor nutritional habits and dietary intake, smoking, sedentary lifestyles, consumption of alcoholic beverages, and economic stressors (3). University faculty and staff are far from immune from these health behaviors and structural conditions that result in chronic diseases from preventable causes.

### Faculty and staff burden of disease

The 2023 American College Association-National Faculty and Staff Health Assessment (ACHA-NFSFA) report reveals that faculty and staff from colleges and universities across the United States maintain a diabetes rate of 16% in men and 13% in women (4), both rates are higher than the national average of 11.6% (5) Faculty and staff members also maintain elevated cholesterol levels (34% of men surveyed and 27% of women), receive less than the 7 h of recommended sleep (46% of men, 45% of women), feel overwhelming anxiety (28% of men, 40% of women), and feel overwhelmed by their responsibilities (48% of men, 64% of women) (4). Tenure-track faculty, especially women and underrepresented minorities, may experience the stresses of and anxiety of promotion tenure-criteria and review differently than their tenured colleagues. These figures may well be the result of an aging academic workforce, in which the average age of a tenure-track faculty member is 49 years, seven years older than the US workforce. Thirteen percent of university faculty are over the age of 65, compared to 6% of the US workforce (6) Universities often employ older faculty since the role of a professor requires many years of advanced training^6^ and the 1986 Discrimination in Employment Act (DEA) prevents academic institutions from mandating retirement ages for faculty and/or staff (7–9). Though age is one possibility, there are a number of potential causes common to academic institutions that may better explain the worsening of university faculty and staff health and wellness in the United States.

### Contributing factors influencing faculty and staff health and wellness

Some of the reasons faculty and staff may not be as healthy as the general population include lack of motivation, poor time management, forgetting about wellness-at-work initiatives, wellness program times that do not fit their schedule, and job responsibilities that make it difficult to attend wellness programming or adhere to a regular meal/exercise schedule (4). A 2025 study of an academic medical center identified factors that help explain the lack of wellness at a personal level within their faculty. These factors included professional and personal life imbalances, feeling disconnected from their colleagues and not supported by the center’s senior leadership (10). Specifically, the time expended due to measures of academic success-the burdensome workload, pressure to publish, responsibilities to students, the need to secure grant funding-contribute to an inability to disconnect from work during personal time (11). Employees in higher education generally have more sedentary job responsibilities than the general public (12), maintain an inadequate dietary intake, and engage in improper eating habits (13) that can result in an obesity-related chronic disease (14). Improper diets and food security are a concern for both faculty and staff members. A qualitative study in 2019 found that faculty may have greater financial resources and access to healthy foods, however the demands of their positions and a lack of available time to shop for or prepare meals prevents them from eating more balanced, nutritious meals, consuming enough food or eating on a regular schedule (15). The staff members, on the other hand, often do not have adequate financial resources, are single heads-of-household and cannot purchase more nutritious food on a regular basis (15). Food insecurity has a direct effect on several obesity-related factors including physical activity and sleep quality and duration, as well as both a direct and indirect effect on body mass index (BMI) in an obesogenic environment (16).

### Existing workplace wellness approaches

In response to employee needs and to improve the wellness of faculty and staff, several universities have instituted either top-down workplace wellness initiatives focused on faculty, students, and staff or invested in cellular phone applications to promote health. One of the most comprehensive and interesting faculty, staff, and student programs is found at a university in Illinois, whose program has been operating for over 40 years (17). Some of the hallmarks of the program is the institutional support demonstrated through the investment in a guiding committee and the inclusion of this wellness program in the university’s strategic plan. The program also includes a campus ambassador program comprised of faculty and students to promote wellness and the allowance of 90-min per week of wellness flex time for all employees to participate in activities (17). A 2024 study also supports the faculty need for some flex time to participate in wellness activities since most staff members do not have the freedom of faculty members to dictate their own schedules (18). The top-down, employee-only programs offered by universities across the United States are generally governed by faculty and leadership teams and include activities such as walking campaigns, mindfulness activities, exercise classes, discounted Weight Watchers memberships, web-based applications, and incentives for participation (19). A few additional pilot studies have been conducted by both faculty and doctoral students. A faculty driven 12-week walking pilot did not achieve the authors’ goal of reducing BMI (20). The mixed-methods study conducted by doctoral students seeking to determine if employees would be more encouraged to utilize staircases to traverse the university buildings when exposed to marketing banners near stairwells found that employee use of staircases (rather than elevators) increased over the course of the 12-week pilot (21).

### Purpose of the study

The purpose of this quasi-experimental study is to determine if faculty and staff consistency of participation, self-efficacy when participating in a program and their comfort beginning a new health and wellness challenge would increase when enrolled in an 8-week broad-based (physical, nutritional, financial, spiritual, and emotional health) health and wellness program designed, developed, planned, marketed, implemented, evaluated, and led wholly by undergraduate public health students. Based on a cursory review of the literature, it is our belief this wholly student-driven program fills a gap in the existing body of literate. Since faculty and staff members are servant leaders, our theory is that they will participate more regularly in a fun, community-building, student-developed campaign. This regular participation is foundational to creating a culture of health at universities and organizations across the nation (22).

## Methods

### Study population and data collection

This was a quasi-experimental study conducted among Faculty and Staff of St. Bonaventure University (SBU). Participants were 18 years and above, employed at SBU, and provided consent by signing the informed consent form. Pre- and post-intervention data were collected using a semi-structured questionnaire administered through Qualtrics. Faculty and staff were also invited to add qualitative clarity for each Likert-scale question on the post-test only. The questionnaire contained 18 questions split into 2 sections-demographic and study-specific questions about comfort, confidence, and current feelings about their engagement in physical activities. Faculty members were invited to contribute qualitative responses to better explain their responses to Likert-scale questions. For each question, faculty and staff were asked about what encouraged/discouraged their participation and asked to recommend changes to the program, based on their experience. By request, the authors will provide a copy of the questionnaire to allow replication studies.

The health and wellness program—branded as the B. O. N. A. S. Challenge—was designed and implemented by 23 undergraduate Public Health majors enrolled in Health Promotion Programming and Program Implementation and Evaluation courses. These students participated through course enrollment rather than as part of a forum or outreach group. Their inclusion was based on course participation, not a competitive selection process. Development followed structured stages: needs assessment, domain prioritization, challenge module creation, pilot testing, final revisions, and program delivery.

The eight weekly challenges were authored by small student teams, each assigned a wellness theme (nutrition, movement, mindfulness, etc.), with faculty mentors providing oversight. A total of 44 participants completed the pre-intervention survey, and 34 completed the post-intervention survey after 8 weeks. Several faculty and staff members did drop out of the program prior to its termination due to scheduling concerns. Since the end of our program approached the end of the academic year, these scheduling concerns seemed appropriate.

### Setting: Cattaraugus County and the rural university context

This study was conducted at St Bonaventure University (SBU) in New York State. St Bonaventure has a student population of roughly two thousand in-person students or distance learners. At the university, there are over 606 full- and part-time faculty and staff members working in various departments. St. Bonaventure University is situated in Cattaraugus County, a rural region in Western New York ranked 56th out of 62 counties in New York State for health outcomes (23). Many university employees live locally and are directly impacted by the county’s high burden of chronic illness and limited healthcare access.

Eighteen percent of adults in the county report fair or poor health—exceeding the statewide average of 14%—and residents average 4.5 physically unhealthy and 5.2 mentally unhealthy days per month (22). Obesity affects 37% of adults, while 26% report no leisure-time physical activity. Access to safe, convenient spaces for physical activity is limited to 68% of the population (23). Risk behaviors and chronic conditions remain elevated with 19% of adults identifying as smokers, compared to the state average of 12% (23). Excessive alcohol use is reported by 22%, and preventable hospital stays total 5,961 per 100,000 Medicare enrollees (23). Socioeconomic pressures exacerbate these health issues: 22% of children live in poverty, and 18% of adults are uninsured (23). Given the challenges faced by New York State, it is reassuring that NYS diabetes rate has declined from 11/4% in 2021 (24) to 10% in 2024 (23). It is equally reassuring to note that Cattaraugus County’s diabetes estimate rivals NYS at 10% in 2024 (23). However, obesity rates in Cattaraugus County are estimated to be 7% higher than the NYS average of 30% (23).

In response, the B. O. N. A. S. Challenge was designed to address modifiable health behaviors within the local context. Its flexible, inclusive structure offered both in-person and virtual opportunities and aimed to reduce participation barriers often faced in rural communities. The program’s structure is aligned with community needs and university wellness goals, demonstrating a sustainable model for worksite health promotion in underserved areas.

### Intervention: the B. O. N. A. S. Challenge

The B. O. N. A. S. (Bonnies Optimizing Nourishment, Achievement, and Strength) challenge was an eight-week wellness initiative targeting St. Bonaventure University’s faculty and staff. Created by undergraduate Public Health students from four courses offered in the Health Education Specialist (HES) concentration, the program functioned as an experiential learning capstone aligned with Health Education Specialist Practice Analysis (HESPA) competencies necessary to sit for the Certified Health Education Specialist (CHES) credentialing exam. Students designed and implemented the intervention with limited faculty input, collaborating weekly with Human Resources and a faculty mentor.

The undergraduate students were only provided the broad guidance to create a faculty and staff health and wellness program focused on increasing the participation of employees in their own health and wellness. The students intentionally selected activities to address diverse domains of wellness—physical, mental, social, and nutritional—based on local health data and stakeholder input. The challenge featured over 20 events, including group fitness sessions, yoga, walking groups, stress-reduction workshops, and healthy cooking demonstrations and 8 additional weekly challenges. The weekly challenges included March Madness (four 1-week daily bodyweight activities), water you waiting for (water drinking challenge), commercial break (bodyweight exercises during commercials), parking challenge (park an additional 6 stalls from intended parking spot), and home cooking challenge (prepare and eat one meal at home daily). A weekly “Lunch and Learn” series tackled topics such as sleep hygiene, hydration, and chronic disease prevention. Supplemental behavior challenges encourage daily habits like gratitude journaling, mindful breathing, or limiting sugar-sweetened beverages.

To encourage sustained engagement, students developed a point-based consistency system tracked through Qualtrics and promoted the initiative via weekly emails and social media. Students enrolled in Public Health marketing took responsibility for campaign branding and communications, while evaluation teams monitored implementation and outcomes. Incentives such as T-shirts and newsletter recognition supported ongoing participation. By emphasizing consistency over perfection and prioritizing accessibility, the Challenge maintained strong faculty and staff involvement while also demonstrating how a student-led project can connect classroom learning with institutional wellness promotion.

#### Dependent variables

The level of self-reported confidence and participation in physical activity and wellness program post-BONAS challenge (post-intervention). Confidence level was not specifically defined for participants, so the measure was more subjective than objective. On-campus participation was recorded via Qualtrics sign-in sheets, however off-campus participation was wholly self-reported. Confidence level was assessed using a six-point Likert scale: “very confident” scored “5”; “somewhat confident” scored “4”; “neither” scored “3”; “not confident” scored “2”; “extremely not confident” scored “1”; “never” scored “0.” Participation level was assessed using a five-point Likert scale: “very regularly” scored “5”; “somewhat regularly” scored “4”; “neither” scored “3”; “not very regularly” scored “2”; “not very regularly at all” scored “1.”

#### Independent variables

The level of self-reported confidence and participation in physical activity and wellness program pre-BONAS challenge (pre-intervention). Confidence level was not specifically defined for participants, so the measure was more subjective than objective. On-campus participation was recorded via Qualtrics sign-in sheets, however off-campus participation was wholly self-reported. Confidence level was assessed using a six-point Likert scale: “very confident” scored “5”; “somewhat confident” scored “4”; “neither” scored “3”; “not confident” scored “2”; “extremely not confident” scored “1”; “never” scored “0.” Participation level was assessed using a five-point Likert scale: “very regularly” scored “5”; “somewhat regularly” scored “4”; “neither” scored “3”; “not very regularly” scored “2”; “not very regularly at all” scored “1.”

#### Statistical analysis

Data collection and editing were conducted manually to detect omissions and to ensure uniform coding. The data was transferred into Excel, and analysis was completed using Statistical Package for the Social Sciences (SPSS) version 29. Frequency tables of the demographic variables pre- and post-intervention were generated. The major categorical variables were coded numerically for analysis. The mean, the mean difference, and the confidence interval of the difference between the pre-and post-intervention were determined. An independent T-test was used to determine the statistical significance of the difference between the dependent (post-intervention) and independent (pre-intervention) variables. The level of significance was determined at a *p*-value < 0.05 with a 95% confidence level (CI).

#### Ethical considerations

The informed consent form was administered to participants before the questionnaire. The questionnaire consisted of two sections, A and B. Section A included questions on participation in wellness programs, and Section B included questions on demographics. Research approval was obtained from the Institutional Review Board of the St Bonaventure University, St Bonaventure, New York. The reference number is 731.

## Results

**Figure 1:**
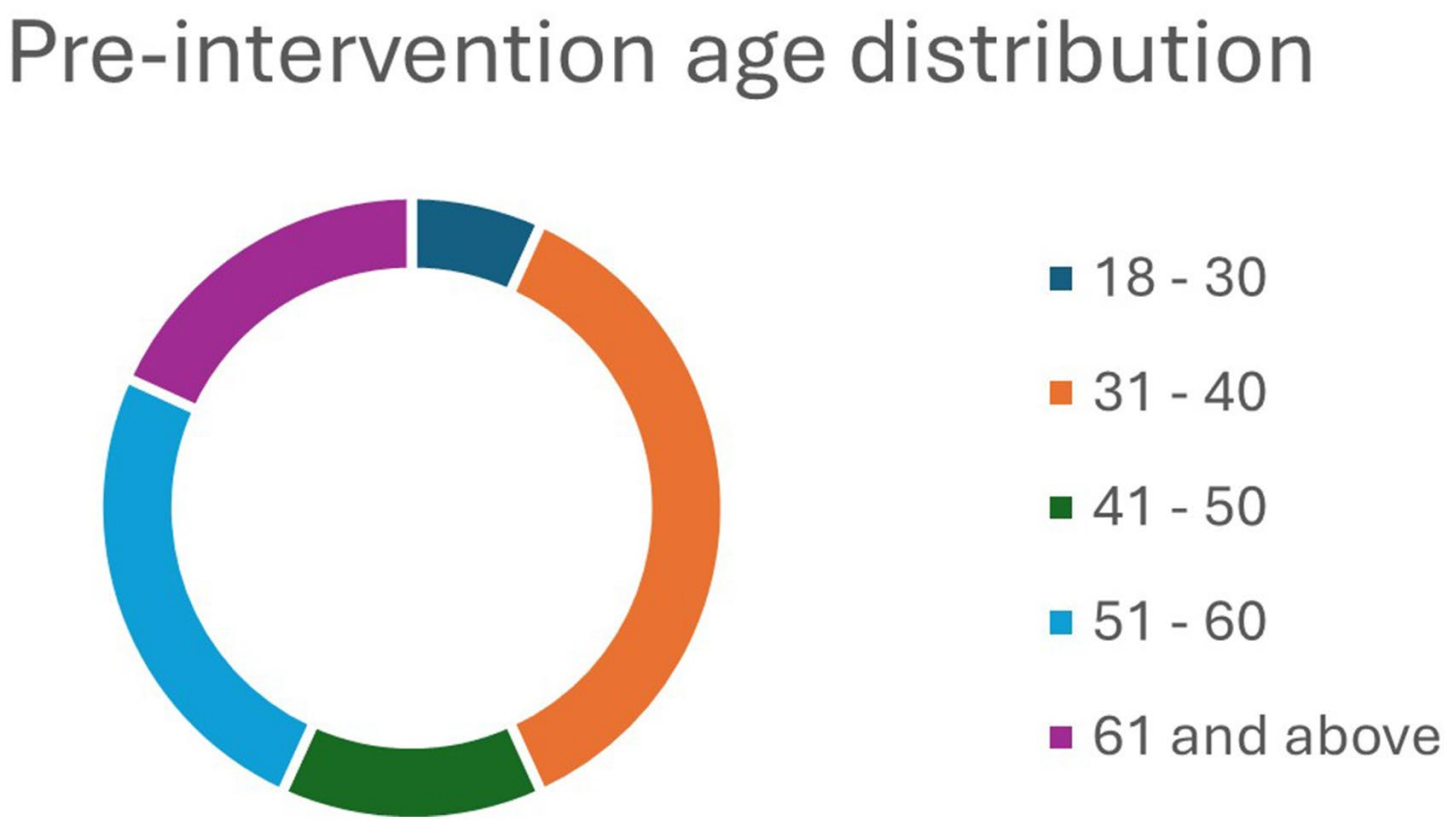
Age distribution pre-intervention.

**Figure 2:**
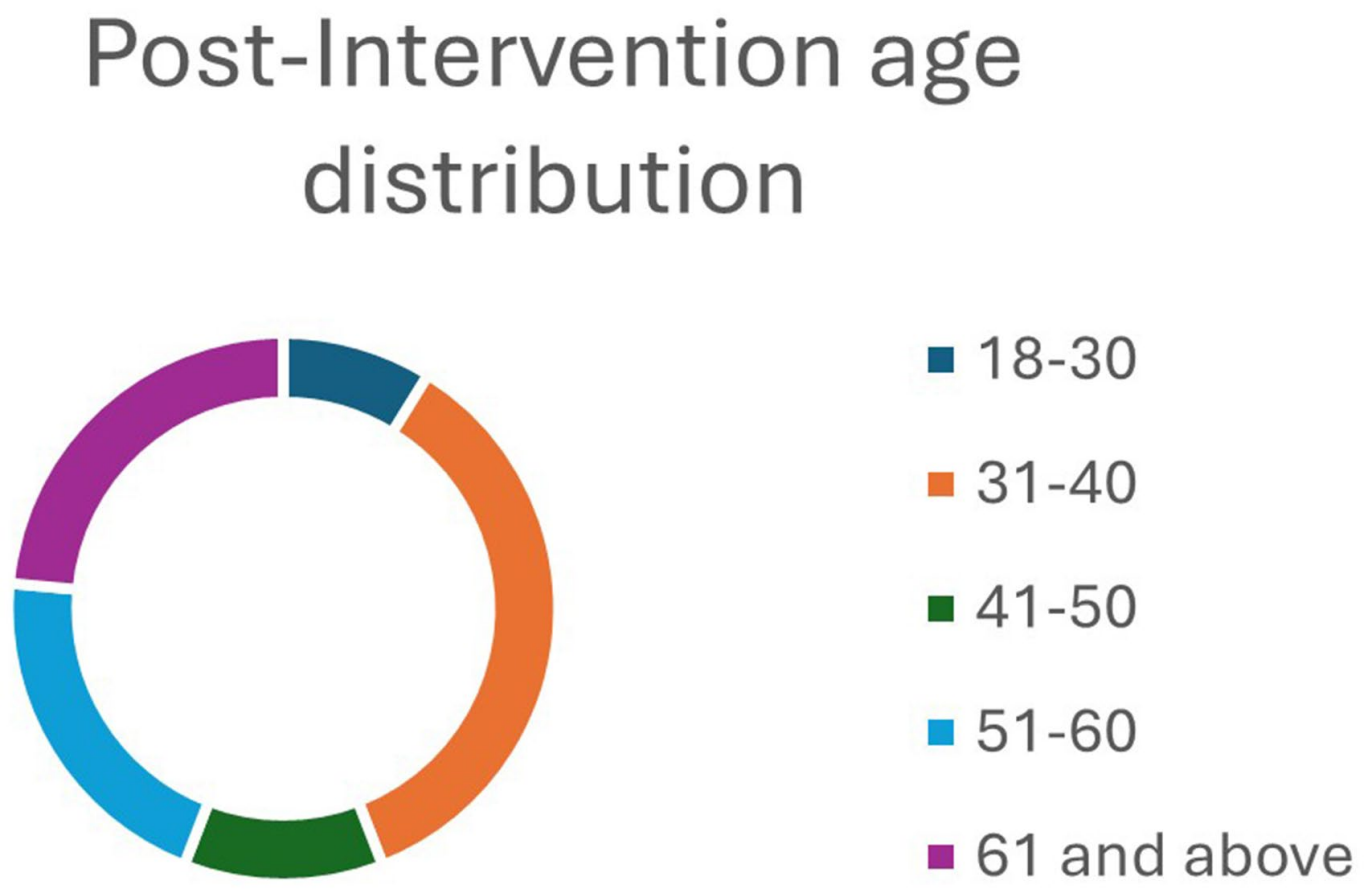
Age distribution post-intervention.

A total of 44 and 34 faculty and staff members responded well to the pre- and post-intervention questionnaire survey, respectively. Most of the participants were females in the pre-intervention (93.18%) and post-intervention (88.24%) groups (Table 1). About two-thirds of the respondents in both groups – pre-intervention group (64.29%) and post-intervention (63.64%)- had a graduate degree. Similarly, more than a third of both pre-intervention (36.36%) and post-intervention (35.29%) groups were within the age group 31–40 years (Table 1). Pre- and post-intervention age breakdowns can be found in Figures 1, 2. Importantly, the age breakdown between groups is to be expected since the program dropouts would alter some of the final demographic information. The predominant race in both groups was white, limiting the ability to generalize these findings to more diverse colleges and universities.

**Table 1:** Demographic variables of staff pre- and post implementation of the wellness program.

Variable	Pre-intervention		Post-intervention	
	Frequency number = 44	Percentage (%)	Frequency number = 34	Percentage (%)
Race				
White	44.00	100.00	33.00	97.06
Black/African American	0.00	0.00	0.00	0.00
Asian	0.00	0.00	1.00	2.94
Hispanic	0.00	0.00	0.00	0.00
Gender				
Female	41.00	93.18	30.00	88.24
Male	3.00	7.31	4.00	11.76
Level of education				
College	13.00	30.95	10.00	30.30
Graduate	27.00	64.29	21.00	63.64
High school or less	1.00	2.38	0.00	0.00
Prefer not to say	1.00	2.28	2.00	6.06
Age (years)				
18–30	3.00	6.81	3.00	8.82
31–40	16.00	36.36	12.00	35.29
41–50	6.00	13.64	4.00	11.76
51–60	11.00	25.00	7.00	20.59
61 and above	11.00	18.18	8.00	23.52

### Comparison of consistency of staff in physical activity in the pre-and post-intervention

In Table 2, there was a statistically significant difference in mean in the participation of staff in the wellness program within SBU and local community pre- and post-BONAS challenge (Mean difference: −0.456, 95% CI: −0882 – −0.048; *p* = 0.029). In a similar vein, there was a statistically significant difference between pre-intervention and post-intervention participation in nutritional programs in SBU and the local community by staff and faculty (Mean difference: −0.472, 95% CI: −0.817 – −0.127; *p* = 0.008). However, there was no statistically significant difference in the mean pre- and post-intervention in comfortability starting a new wellness program and confidence participating in a new wellness program by staff and faculty at the university with *p* = 0.628 and 0.593, respectively. Faculty and staff members, who either provided a qualitative response or contacted one of the authors to share their feedback indicated that their overall participation improved, thanks to the regular marketing for the B. O. N. A. S. Challenge, calendar invitations to participate, post-event marketing, weekly newsletters and from seeing other participants adorned in their team t-shirts.

**Table 2:** Comparison of staff’s consistency in physical activity and wellness programs pre- and post- intervention.

Variables	Mean	*T*- test	Mean difference	Confidence interval of the difference	*P*- value
Engagement in physical activity					
Pre-intervention	1.378				
Post-intervention	1.471	−0.459	**−0.093**	−0.496 – 0.310	0.648
Participation in community physical activity					
Pre-intervention	0.681				
Post-intervention	1.147	−2.224	−0.465	−0.882 – 0.048	0.029
Participation in nutritional program					
Pre-intervention	0.204				
Post-intervention	0.676	−2.742	−0.472	−0.817 – 0.127	0.008
Schedule allowance for wellness program					
Pre-intervention	1.349				
Post-intervention	1.424	−0.362	−0.075	−0.480 – 0.339	0.716
Schedule prevents eating balanced meal					
Pre-intervention	0.772				
Post-intervention	0.647	0.602	0.126	−0.290 – 0.541	0.549
Comfortable starting a new wellness program					
Pre-intervention	1.773				
Post-intervention	1.706	0.493	0.067	−0.203 – 0.337	0.628
Confidence starting a new wellness program					
Pre-intervention	1.727				
Post-intervention	1.647	0.537	0.08	−0.217 – 0.377	0.593

## Discussion

The current study focused on the consistency of faculty and staff participation when engaged in a wholly student-led, −designed, −developed, −marketed, −implemented, and -evaluated health and wellness campaign. The study was conducted at a small, private university housed within a rural county in Western New York State on the East Coast of the United States. The surrounding county suffers from poor health outcomes and maintains rates of diabetes, obesity, poverty, food insecurity, and physical inactivity higher than both the state and national averages (23).

The results derived from *t*-tests utilizing the Likert-scale Qualtrics pre- and post-test data indicate that there was both a significant increase in faculty and staff consistency our wellness program and in community-based wellness activities (*p* = 0.029) and in nutritional programming from within and without the university setting (*p* = 0.0008). T-tests were preferable to use for this analysis, as paired t-test analysis would have required the same participants complete both the pre- and post-intervention test. Since the study allowed rolling participation, matched participation was more complicated to solicit. Our program was focused on consistency, so we chose not to limit faculty and staff participation to events held on-campus and broadened the arena to allow consistency tracking points for activities off-campus. Community-based activities included visits to local gyms, dance classes, running clubs, etc. and consistency from community activities was self-reported by participants.

This consistent behavior is best explained by both habit training and nudging theory. Habit training and habit theory, often utilized by occupational therapists to retrain patients to perform the activities of daily living, focuses on performing and reminding patients of a task to be performed until the habituation reduces the amount of conscious thought needed to perform these context- and environment-dependent behaviors (25–27). The context necessary for a behavior to become a habit also involves the choices available to a participant. If a participant can choose between a physical activity, nutritional activity or drinking more water daily, they may choose the easiest of the behaviors or try each but not develop the habituation needed for said behavior to become routine (28). A study conducted in the UK focused on how habits are formed and determined that the variable length of time necessary to convert a repeated behavior into a habit ranged from 18–254 days (28, 29). Nudging theory and nudging interventions, on the other hand, focus on promoting better health behavior changes through gentle reminders, signage, regular health marketing, and product positioning and placement (30). Grocery stores often rely on nudging by placing items at the end of rows of grocery to nudge a buyer with limited time to shop into an impulse purchase (30).

This study found that participant comfort starting and confidence when participating in a health and wellness campaign did not meet the threshold of significance. Though very few of the participants elected to provide written qualitative feedback, some of the possible reasons for this result could include the nature of some of the campaign’s activities, the time management skills of the participants, the timing of the intervention which neared finals week and the end of the academic year, holding indoor activities as the weather improved, timing of the events offered, discomfort when engaging in physical activities with colleagues, body dysmorphia and self-image concerns, and fear of the unknown. The aforementioned reasons many faculty and staff members became inconsistent in their on-campus participation are from conversations with the authors initiated by participants. In retrospect, participant confidence and comfort starting a program could have improved had elements of social support been added to the program. Social support has been associated with increased self-efficacy when engaged in physical activities, in particular (31). In addition, employing a targeted motivation-focused, empowering campaign strategy may have improved participant comfort when beginning this program. Higher self-efficacy has been associated with reduced barriers to physical activity and positive outcome expectations (31). Incorporating self-imagery activities into this wellness campaign may have influenced activity and motivation to perform. Self-imagery is a process in which participants can imagine themselves performing an activity successfully (32). Without the adequate qualitative responses from the post-intervention survey, the reasons listed above are from anecdotal observations and conversations with participants.

This study is not without its limitations. The current paper was not originally designed as research, as it was developed as a student learning opportunity. As a result and given the truncated lead time to begin the program, the quasi-experimental study lacks a control group and relied on self-reported data. Since the study was performed at a small, private 4-year university and the sample size was limited, the results of this study may not be generalizable to larger, public institutions of higher learning nor would these results be indicative of the success of a faculty and staff health and wellness program in k-12 school systems. More so, there is a possibility of social desirability bias as the questionnaire was self-administered. Additionally, the study population was largely Caucasian and female, which further limits the ability to generalize our findings. Another limitation was the fragmented funding sources and the lack of institutional support by the university’s senior leadership. The addition of qualitative queries found in the post-intervention survey would have been more useful had we added qualitative response options to the pre-test. In addition, coding participants and their responses would have allowed for deeper analysis of the qualitative data.

Some of the strengths of this study include its novelty and that the totality of this campaign was wholly student-designed and -driven. In addition, this program was dedicated to the improvement of faculty and staff wellness, as we posited that faculty and staff members serve as examples for the students and would be more likely to model these behaviors when engaged in a student-driven activity campaign. Upon a cursory review of the literature, there are few to no other programs like this that are wholly driven by undergraduate students to improve the wellbeing of university faculty and staff.

## Conclusion

The current study focused on improving the consistency of participation by faculty and staff in a student-led, −developed, −designed, and implemented health and wellness campaign. Our findings indicate that consistency of faculty and staff participation increased significantly both on- and off-campus over the course of the 8-week campaign. The authors’ theorized that faculty and staff would continue to participate in a program designed by students since university staff members often serve as servant leaders and examples to the student body.

## Data Availability

The raw data supporting the conclusions of this article will be made available by the authors, without undue reservation.
